# *Glaesserella parasuis* serotype 4 HPS4-YC disrupts the integrity of the swine tracheal epithelial barrier and facilitates bacterial translocation

**DOI:** 10.1186/s13567-021-01005-w

**Published:** 2021-10-21

**Authors:** Qing Wang, Xiaojing Chang, Mingxing Liu, Qi Lu, Meng Zhu, Huixing Lin, Hongjie Fan

**Affiliations:** 1 Anhui Province Key Laboratory of Veterinary Pathobiology and Disease Control, College of Animal Science and Technology, Anhui Agricultural University, Hefei, 230036 China; 2 MOE Joint International Research Laboratory of Animal Health and Food Safety, College of Veterinary Medicine, Nanjing Agricultural University, Nanjing, 210095 China; 3 Jiangsu Co-Innovation Center for the Prevention and Control of Important Animal Infectious Diseases and Zoonoses, Yangzhou University, Yangzhou, 225009 China

**Keywords:** *Glaesserella parasuis*, swine tracheal epithelial cells, tight junction proteins, proinflammatory cytokines

## Abstract

*Glaesserella parasuis* (*G. parasuis*) is a commensal bacterium in the upper respiratory tract of pigs that can also cause the swine Glässer disease, which induces an intensive inflammatory response and results in significant economic losses to the swine industry worldwide. *G. parasuis* can cause disease through infection of the respiratory tract, resulting in systemic infection, but the mechanism is largely unknown. Recently we showed that *Glaesserella parasuis* serotype 4 (GPS4) increased swine tracheal epithelial barrier permeability, resulting in easier bacterial translocation. Tight junction proteins (TJ) play a crucial role in maintaining the integrity and impermeability of the epithelial barrier. GPS4 decreased the expression of the TJ ZO-1 and occludin in swine tracheal epithelial cells (STEC). Furthermore, the proinflammatory cytokines IL-6, IL-8 and TNF-α were significantly upregulated in GPS4-infected STEC, and both the MAPK and NF-κB signaling pathways were activated and contributed to the expression of TNF-α. We demonstrate that the production of proinflammatory cytokines, especially TNF-α, during GPS4 infection was involved in barrier dysfunction. Additionally, animal challenge experiments confirmed that GPS4 infection downregulated TJ in the lungs of piglets and induced a severe inflammatory response. In general, *G. parasuis* infection downregulated the expression of TJ and induced massive secretion of proinflammatory cytokines, resulting in epithelial barrier disruption and favoring bacterial infection. This study allowed us to better understand the mechanism by which *G. parasuis* crosses the respiratory tract of pigs.

## Introduction

*Glaesserella parasuis* (*G. parasuis*), the causative agent of Glässer disease, is a Gram-negative opportunistic bacterial pathogen that colonizes the upper respiratory tract of pigs. *G. parasuis* infection induces severe systemic inflammatory reactions characterized by fibrinous polyserositis, pneumonia, arthritis and meningitis, resulting in extensive economic losses for the pig industry [1, 2]. To date, 15 serotypes of *G. parasuis* have been defined, in addition to nontypeable isolates. Serotypes 4 and 5 are the most prevalent serovar isolates from pigs with clinical signs of Glässer disease in China [3]. Secondary or concurrent *G. parasuis* infection with other pathogens such as porcine reproductive and respiratory syndrome virus (PRRSV) or swine influenza virus, remains an epidemic, leading to increased morbidity and mortality, and resulting in more severe challenges to the control of *G. parasuis* infection [4, 5].

As a pathogen that can cause infection through the respiratory tract, *G. parasuis* has to break through the respiratory epithelial barrier to enter the bloodstream and induce systemic infection. The respiratory epithelium is an important barrier to defend against harmful particles and inhaled pathogens, and barrier failure of the respiratory epithelium contributes to the pathogen invasions [6, 7]. The epithelial barrier is mainly composed of the apical junctional complex, composed of tight junctions, adhesion junctions and desmosomes [8, 9]. Tight junction proteins (TJ), including ZO-1, occludin and claudins, play a major role in maintaining the barrier function of epithelial cells [7]. Some studies have demonstrated that respiratory pathogens can disrupt epithelial TJ and damage barrier function [10, 11]. For example, *Streptococcus pneumoniae* and *Haemophilus influenzae* infection cause downregulation of TJ in human airway epithelial cells and induce disruption of epithelial barrier integrity [11]. Additionally, *Mycoplasma hyopneumoniae* reduces ZO-1 expression and disrupts the integrity of the porcine airway epithelial barrier [12]. However, there are few studies on the effects of *G. parasuis* on TJ expression and respiratory epithelial barrier integrity [13, 14]. The aim of the study is to investigate how *G. parasuis* disrupts the respiratory epithelial barrier to cause systemic infection.

Numerous studies have reported that excessive proinflammatory cytokine production can damage the integrity of the epithelial barrier [15]. Petecchia et al. reported that exposure to the cytokines TNF-α, IL-4 and IFN-γ significantly reduced the expression of the TJ ZO-1 and occludin, damaging the human airway epithelial barrier [16]. In addition, TNF-α secreted by pig tracheal epithelial cells damage TJ and disrupt the epithelial barrier; TNF-α can also promote IL-6 and IL-8 secretion in epithelial cells [13]. Previous studies have shown that *G. parasuis* infection evokes a strong inflammatory response in the host, and the excessive release of cytokines can cause lung damage [17]. However, whether this excessive inflammatory response may have a destructive effect on the integrity of the epithelial barrier is still unclear.

In this study, swine tracheal epithelial cells (STEC) were used to explore the effects of *G. parasuis* on the expression of TJ and permeability of the tracheal epithelial barrier. The production of proinflammatory cytokines and related signaling pathways were further evaluated. The effects of secreted cytokines on the permeability of the epithelial barrier were assessed. In addition, *G. parasuis-*induced disruption of TJ and a severe inflammatory response were verified through piglet challenge experiments.

## Materials and methods

### Bacterial strain and cell line

The GPS4 strain used in this study, HPS4-YC, was isolated from a diseased pig in Jiangsu, China, in 2016. Bacteria were cultured in trypticase soy broth (TSB, BD, USA) or on trypticase soy agar (TSA) supplemented with 5% (v/v) fetal bovine serum (FBS, Gibco, USA) and 50 μg/mL nicotinamide adenine dinucleotide (NAD, Biosharp, China).

Immortalized swine tracheal epithelial cells (STEC) were cultured in Dulbecco modified Eagle medium (DMEM, Gibco, USA) supplemented with 10% (v/v) FBS (Gibco, USA) at 37 °C and 5% CO_2_. Subsequently, cells were digested with trypsin, suspended in culture medium, distributed into cell culture plates, and incubated until the cells were confluent.

### Giemsa staining

STEC were cultured on sterile glass slides until completely confluent. GPS4 strain HPS4-YC was grown in supplemented TSB overnight at 37 °C and 180 rpm. Bacteria were harvested by centrifugation at 5000 × *g* for 5 min, washed three times with phosphate buffered saline (PBS) and resuspended in DMEM without FBS. Cells were infected with HPS4-YC with different multiplicity of infection (MOI) values of 10, 100 or 1000. Three parallel samples were set for each group. After incubation for 2 h at 37 °C and 5% CO_2_, the cells were washed three times with PBS and fixed with 4% paraformaldehyde for 30 min. The slides were then dyed with Giemsa staining solution (Beyotime, China). After washing away the dye solution, the slides were dried and observed under an optical microscope.

### Adherence and invasion assays

STEC were distributed into 24-well plates and incubated at 37 °C in 5% CO_2_ until the cells were confluent. Cells were then inoculated with HPS4-YC at different MOI of 10, 100 or 1000. Plates were centrifuged at 800 × *g* for 10 min. After 2 h of incubation at 37 °C with 5% CO_2_, cells were washed three times with PBS to remove nonadherent bacteria. For the adhesion assay, the cells were lysed with double-distilled water and diluted appropriately with PBS. The number of adherent bacteria was determined by spreading on supplemented TSA. For the invasion assay, extracellular bacteria were killed by the addition of DMEM containing 100 µg/mL gentamicin (Solarbio, China) and 5 μg/mL penicillin G (Solarbio, China) for an additional 1 h. Antibiotic-treated cells were washed three times in PBS, lysed with double-distilled water, and diluted appropriately with PBS before spreading on supplemented TSA to determine bacterial counts. Assays were performed in triplicate and repeated three times.

### Measurement of cell viability

An Enhanced Cell Counting Kit-8 (CCK-8, Beyotime, China) was used to assess the viability of STEC following the manufacturer’s protocols. Briefly, STEC were seeded at 2.0 × 10^3^ cells/well in 96-well plates and then infected with HPS4-YC at different MOI of 10, 100 or 1000 for 6, 12 and 24 h. After incubation for the designated time, 10% (v/v) CCK-8 solution was added and incubated at 37 °C for 1 h in darkness. Wells without cells were assayed as background. Absorbance at 450 nm was detected using an enzyme-linked immunosorbent assay reader. Assays were performed with four replicates and repeated as three independent experiments.

### Determination of epithelial barrier integrity

According to our previous study, an in vitro tracheal epithelial barrier model was constructed with STEC using polytetrafluoroethylene 3 μM pore-size membrane transwells (Corning, USA) [18]. Transwells containing STEC were uninfected or infected with HPS4-YC (MOI 100) for 4, 6, 8, 12, 18 and 24 h. The transepithelial electrical resistance (TEER) was measured with a Millicell ERS-2 electrical resistance system (Millipore, USA). Moreover, FITC-conjugated dextran 4000 (Sigma, USA) was used to measure the paracellular permeability of the tracheal epithelial barrier infected with HPS4-YC for 6, 12, 18 and 24 h. After experimental treatment, the media in the upper and lower chambers were removed; the basolateral medium was replaced with fresh DMEM, and then DMEM containing 1 mg/mL 4 kDa FITC-conjugated dextran was added to the apical chamber of the inserts. One hour later, 100 μL of sample media from the basolateral chambers was collected and tracer concentrations were quantified by Tecan Infinite 200 PRO (Tecan, Switzerland). Additionally, STEC were uninfected or infected with HPS4-YC for 24 h, and the supernatant was harvested and filtered with a 0.22 μm filter to remove suspended bacteria before being added to the upper chambers for another 12 h or 24 h. Additionally, recombinant human TNF-α (Cell Signaling Technology, USA) was added to the upper chambers at concentrations of 0.1, 1, or 10 ng/mL and incubated for 24 h. TEER and FITC-dextran were measured as described above. All assays were performed in triplicate and repeated three times.

### HPS4-YC translocation assay

Experiments were performed when an in vitro tracheal epithelial barrier model was constructed. Transwells containing STEC were infected with HPS4-YC at an MOI of 100 for 4, 6, 8, 10, 12, and 14 h. The medium in the basolateral chamber of the transwells was collected and spread on supplemented TSA plates to count bacterial colonies. Assays were performed with four replicates and repeated as three independent experiments.

### Western blotting

STEC grown in 24-well plates were infected with HPS4-YC at an MOI of 100 for the designated time. Cells were lysed in RIPA buffer (Proteintech, China) and centrifuged, and the supernatant was collected. The lysate proteins were boiled with 5 × SDS-PAGE Sample Loading Buffer (KeyGEN Biotech, Nanjing, China) for 10 min. Then, the proteins were separated on a gel by SDS-PAGE and transferred to a 0.22 μm polyvinylidene fluoride (PVDF) membrane. Membranes were blocked in 5% skimmed milk for 2 h at 37 °C and incubated with specific primary antibodies at 4 °C overnight. Then, membranes were incubated with HRP-goat anti-mouse IgG H&L (1:5000, Proteintech, China) or HRP-goat anti-rabbit IgG H&L (1:5000, Proteintech, China) for 1 h at 37 °C. Protein bands were incubated with an ECL chemiluminescence detection kit (Vazyme, China) and visualized using a chemiluminescence image analysis system (Tanon, China). The protein band intensities were quantified by ImageJ software, and β-actin was used as a control. The primary antibodies used were as follows: anti-ZO-1 mouse mAb (1:500, Invitrogen, USA), anti-occludin mouse mAb (1:1000, Invitrogen, USA), anti-phospho-p38 rabbit mAb, anti-p38 rabbit mAb, anti-phospho-ERK rabbit mAb, anti-ERK rabbit mAb, anti-phospho-JNK rabbit mAb, anti-JNK rabbit mAb, anti-phospho-NF-κB p65 rabbit mAb, anti-NF-κB p65 rabbit mAb, anti-phospho-IκBα rabbit mAb, anti-IκBα rabbit mAb (1:1000, Cell Signaling Technology, USA), and anti-β-actin mouse mAb (1:5000, Proteintech, China).

### Quantitative reverse transcription PCR (qRT-PCR) assays

Total RNA was extracted from STEC using TRIzol reagent (TaKaRa, Japan). cDNA was synthesized using HiScript Q RT SuperMix for qPCR (+gDNA wiper) (Vazyme, China). qRT-PCR were performed according to the instructions of ChamQ Universal SYBR qPCR Master Mix (Vazyme, China) on the ABI StepOne Real-Time PCR System (Applied Biosystems, USA). The *GAPDH* gene was used as an internal control. All primers are shown in Table 1, and relative quantification compared to the uninfected cells was calculated based on the 2^−ΔΔCt^ method [19]. All qRT-PCR tests were performed in triplicate.

**Table 1:** **qRT-PCR primers**

Gene	Forward	Reverse
ZO-1	GGGTGTTGAGCTCCATAGAAA	GTCTCGGCAGACCTTGAAATA
occludin	CGGATTCTGTCTATGCTCGTTAT	TAGCCCATACCACCTCCTATT
IL-6	GGAGACCTGCTTGATGAGAATC	CAGCCTCGACATTTCCCTTAT
IL-8	TTCTGCAGCTCTCTGTGAGGC	GGTGGAAAGGTGTGGAATGC
TNF-α	CCTACTGCACTTCGAGGTTATC	ACGGGCTTATCTGAGGTTTG
GAPDH	GATGCTGGTGCTGAGTATGT	GGCAGAGATGATGACCCTTT

### Immunofluorescent staining of ZO-1 and occludin

STEC were uninfected or infected with HPS4-YC for 12 h or 24 h, then cells were fixed with 4% paraformaldehyde (Beyotime, China) for 15 min. After being blocked with 1% BSA at 37 °C for 1 h, samples were incubated with primary antibodies against ZO-1 (5 µg/mL, Invitrogen, USA) and occludin (5 µg/mL, Invitrogen, USA) at 4 °C overnight. Then, the samples were incubated in FITC goat anti-mouse IgG (1:200, Abbkine, China) at 37 °C for 1 h in the dark. Finally, nuclei were stained with DAPI (Beyotime, China). All the specimens were examined using a Zeiss laser scanning microscope (Carl Zeiss, Germany). Assays were repeated as three independent experiments.

### Inhibition assay

To explore the roles of mitogen-activated protein kinases (MAPK) and nuclear factor-kappa B (NF-κB) signaling pathways in cytokine production, STEC were pretreated with different inhibitors for 1 h. The infection time of HPS4-YC was 12 h. The inhibitors used were as follows: SB203580 (p38 inhibitor, MCE, USA), U0126 (ERK inhibitor, MCE, USA), SP600125 (JNK inhibitor, MCE, USA), and BAY11-7082 (NF-κB inhibitor). The concentration of inhibitor used was 10 μM, based on a previously published reference [20]. STEC treated with equal volumes of DMSO served as controls.

### Animal challenge experiments

Five three-week-old female piglets were selected from a healthy herd. All piglets were confirmed to be seronegative for PCV2, PRRSV, *G. parasuis*, *Streptococcus suis* serotype 2 and *Actinobacillus pleuropneumoniae*. One week later, piglets were randomly divided into two groups: the HPS4-YC-infected group (*n* = 3) and the control group (*n* = 2). Piglets in the HPS4-YC-infected group were challenged intranasally (1 mL) and intraperitoneally (2 mL) with HPS4-YC (4.0 × 10^9^ CFU/mL) on the same day. The infection methods and doses were determined from previously published references[17, 21]. Blood samples were taken at 3, 5 and 7 days post-inoculation (dpi) with HPS4-YC for serum isolation. Piglets were necropsied at 7 dpi, and lung samples were collected for further analysis. The animal experiments were approved by the Ethical Committee for Animal Experiments of the Nanjing Agricultural University (NJAU. No20210510064) and were in accordance with the guidelines of the Animal Welfare Council of China.

Total protein and RNA were extracted from equal amounts of lung tissues from different areas of the lungs according to the instructions of the Whole Cell Lysis Assay (Solarbio, China) and Total RNA Kit I (OMEGA, USA). Western blotting and qRT-PCR assays were performed to evaluate the expression of ZO-1 and occludin. Levels of cytokines IL-6, IL-8 and TNF-α in the serum samples or culture supernatant of STEC were assessed using commercial ELISA kits (Fankew, China). Hematoxylin and eosin (H&E) staining of the lungs and immunofluorescence staining for ZO-1 and occludin were completed by Wuhan Servicebio Technology Co., Ltd.

### Statistical analysis

The results in the study were recorded as the mean ± standard deviation (SD) and analyzed and graphed using GraphPad Prism 8. Statistical differences were assessed using Student *t* test and one-way analysis of variance (ANOVA) with Tukey test. A value of *P* < 0.05 was considered significant.

## Results

### Infection of HPS4-YC in STEC

**Figure 1:**
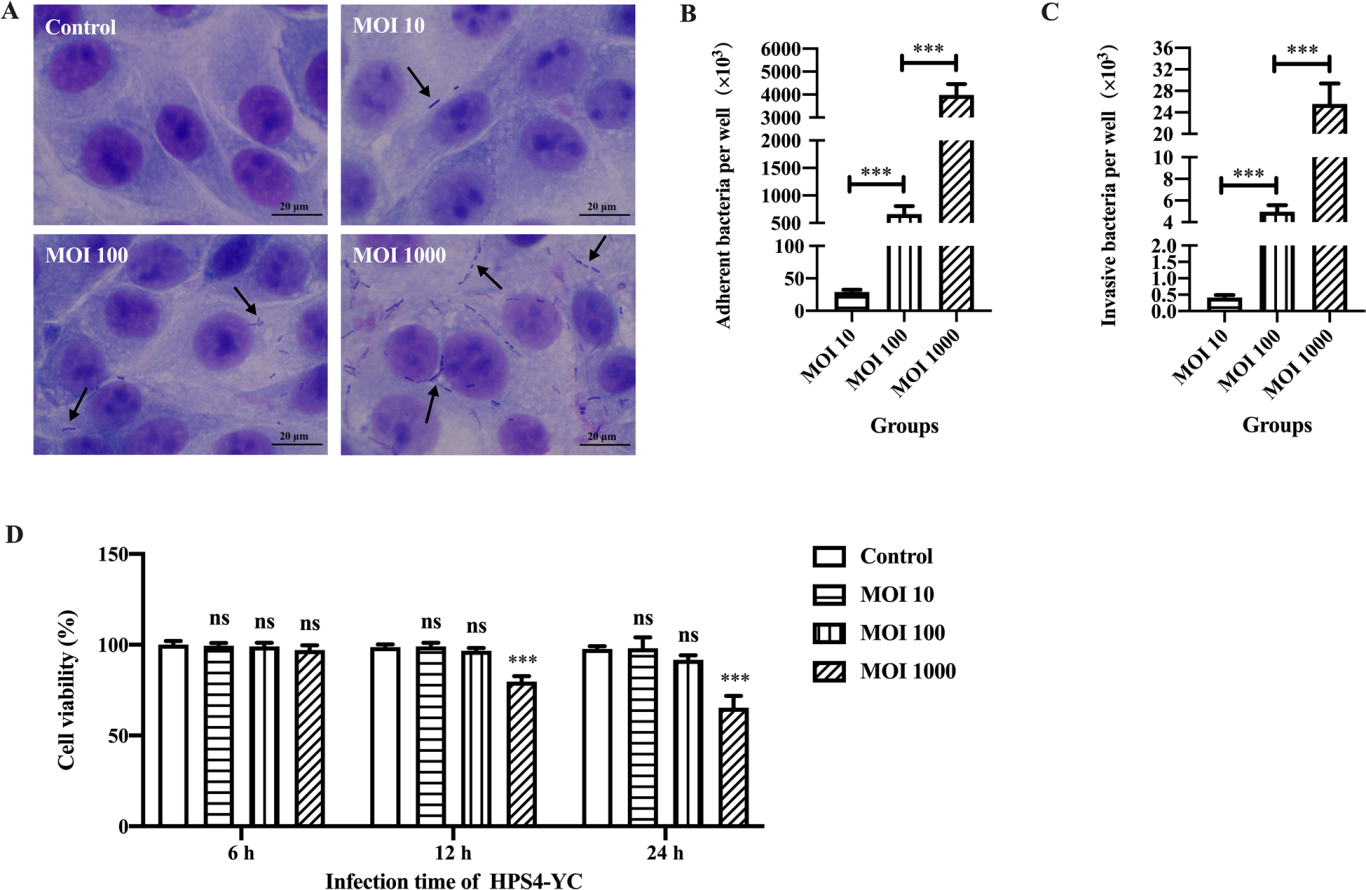
**HPS4-YC infection in STEC.** **A** Giemsa staining of HPS4-YC infecting STEC (100×). STEC were uninfected or infected with HPS4-YC at an MOI of 10, 100 or 1000 for 2 h. The black arrows point to the bacteria present. Scale bar, 20 μm. **B**, **C** Adherence and invasion assays. STEC were infected with HPS4-YC at different MOI for 2 h. The number of adherent and invasive bacteria per well was counted. **D** Cell viability of STEC were assessed by CCK-8. The results represent the means ± SD of three independent experiments. Significant differences in B and C were analyzed using Student* t* test. Significant differences in D were analyzed using one-way ANOVA. ns: not significant; ***P < 0.001.

To establish an in vitro GPS4-infected cell model, the susceptibility of STEC infected by HPS4-YC was evaluated. Giemsa staining results show that HPS4-YC had a potential to infect STEC, and that the number of bacteria observed increased with increasing MOI (Figure 1A). To quantify the number of HPS4-YC adherent and invading STEC, adherence and invasion assays were performed. As shown in Figures 1B, C, when the MOI of HPS4-YC was 1000, the adherent and invasive bacteria were significantly higher (*p* < 0.001) than that at an MOI of 100, when the MOI of HPS4-YC was 100, the adherent and invasive bacteria were significantly higher (*p* < 0.001) than that at an MOI of 10, with the number of bacteria that adhered and invaded STEC being dose-dependent; this result was consistent with the results of Giemsa staining.

Additionally, cell viability was evaluated to determine the optimal MOI of HPS4-YC in vitro. The results show that there was no significant cytotoxicity to infected cells when the MOI of HPS4-YC were 10 and 100 within 24 h. However, when the MOI increased to 1000, the cell viability was significantly decreased at 12 h and 24 h (*p* < 0.001) compared with the control group (Figure 1D). Therefore, an MOI of 100 was selected as the most appropriate MOI in subsequent experiments.

### HPS4-YC infection increased permeability of the tracheal epithelial barrier, allowing more rapid translocation of bacteria across epithelial monolayers

**Figure 2:**
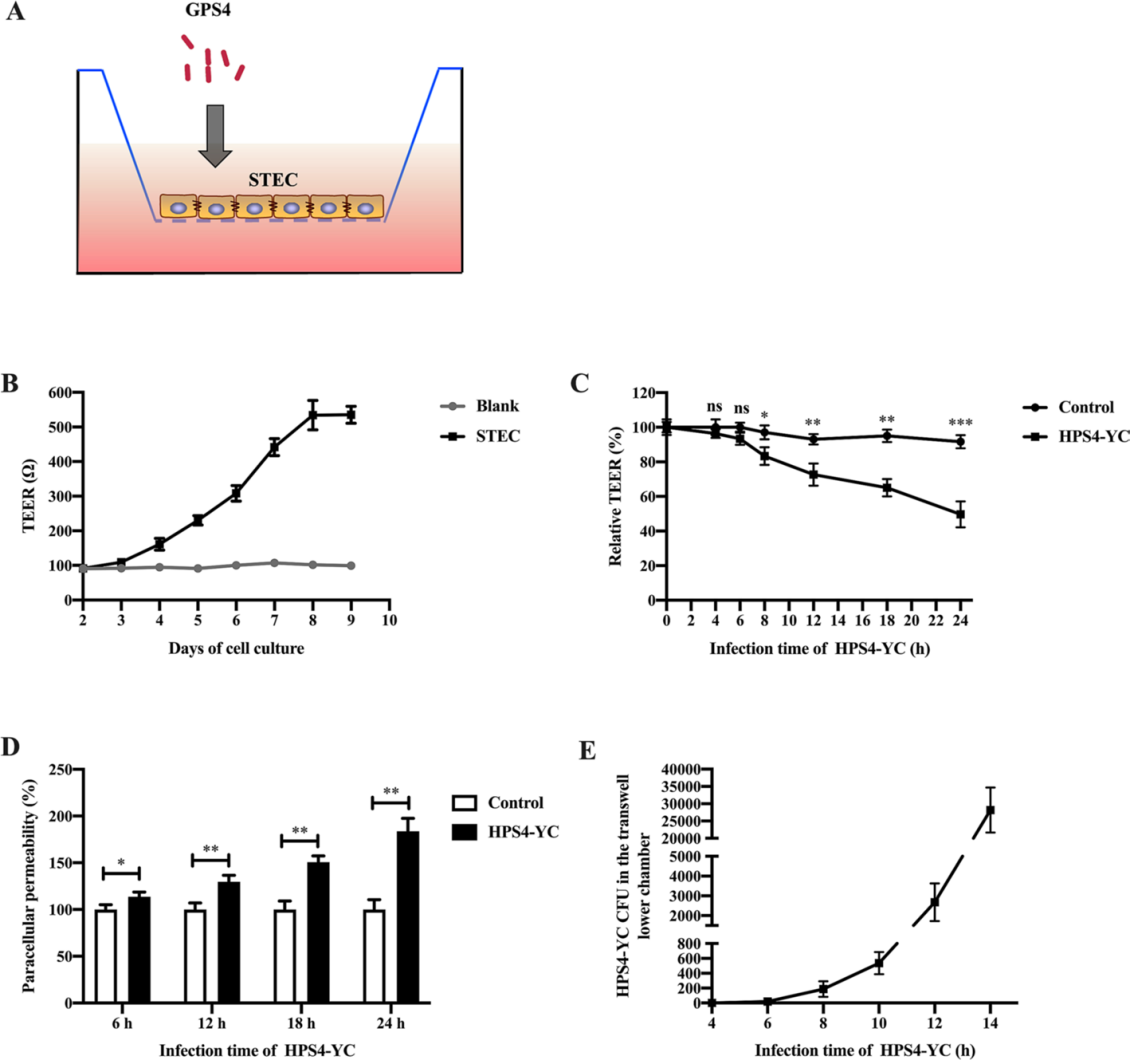
**HPS4-YC infection increased tracheal epithelial barrier permeability.** **A** In vitro model of tracheal epithelial barrier. STEC were cultured in a transwell, GPS4 was added to the upper chamber. **B** Construction of an in vitro tracheal epithelial barrier model. **C** HPS4-YC infection decreased the TEER of the epithelial barrier. STEC monolayers were uninfected or infected with HPS4-YC for different times, and TEER was measured. **D** Measurement of paracellular permeability. The epithelial barrier integrity was analyzed by monitoring FITC-conjugated dextran crossing the transwell chamber. Paracellular permeability is shown relative to the uninfected permeability. **E** CFU of HPS4-YC across the epithelial barrier model were counted. The results are shown as the mean ± SD of three separate experiments. Significant differences in B and C were determined using the Student *t* test. ns: not significant; *, *P* < 0.05; **, *P* < 0.01; ***P < 0.001.

To evaluate the influence of HPS4-YC infection on the paracellular permeability of STEC, an in vivo tracheal epithelial barrier model was established (Figure 2A). The TEER value is an indicator for assessing the integrity of the cell barrier [22]. The stabilization of TEER indicates the success of the construction (Figure 2B). Compared with the control group, HPS4-YC infection significantly decreased the TEER at 8, 12, 18 and 24 h after infection, and there was no significance at 4 h and 6 h post-infection of HPS4-YC (Figure 2C), indicating that HPS4-YC infection gradually reduced the TEER value.

Furthermore, we investigated the impact of HPS4-YC on the permeability of the tracheal epithelial barrier by measuring the concentration of FITC-dextran crossing the transwell chamber. Relative to the uninfected control group, the permeability of the tracheal epithelial barrier in the HPS4-YC infection group was significantly increased in a time-dependent manner, as shown in Figure 2D. The results above indicate that HPS4-YC infection induced increased permeability and destroyed the integrity of the tracheal epithelial barrier.

The number of HPS4-YC penetrating the epithelial barrier model was counted at different times post-infection. As shown in Figure 2E, HPS4-YC crossed the in vitro tracheal epithelial barrier constructed with STEC after incubation for 8 h, and the number of penetrated bacteria increased sharply with increasing infection time. This result further confirmed that HPS4-YC induced the breakdown of the epithelial barrier and contributed to the translocation of the bacterial pathogen GPS4.

### HPS4-YC caused TJ disruption in STEC

**Figure 3:**
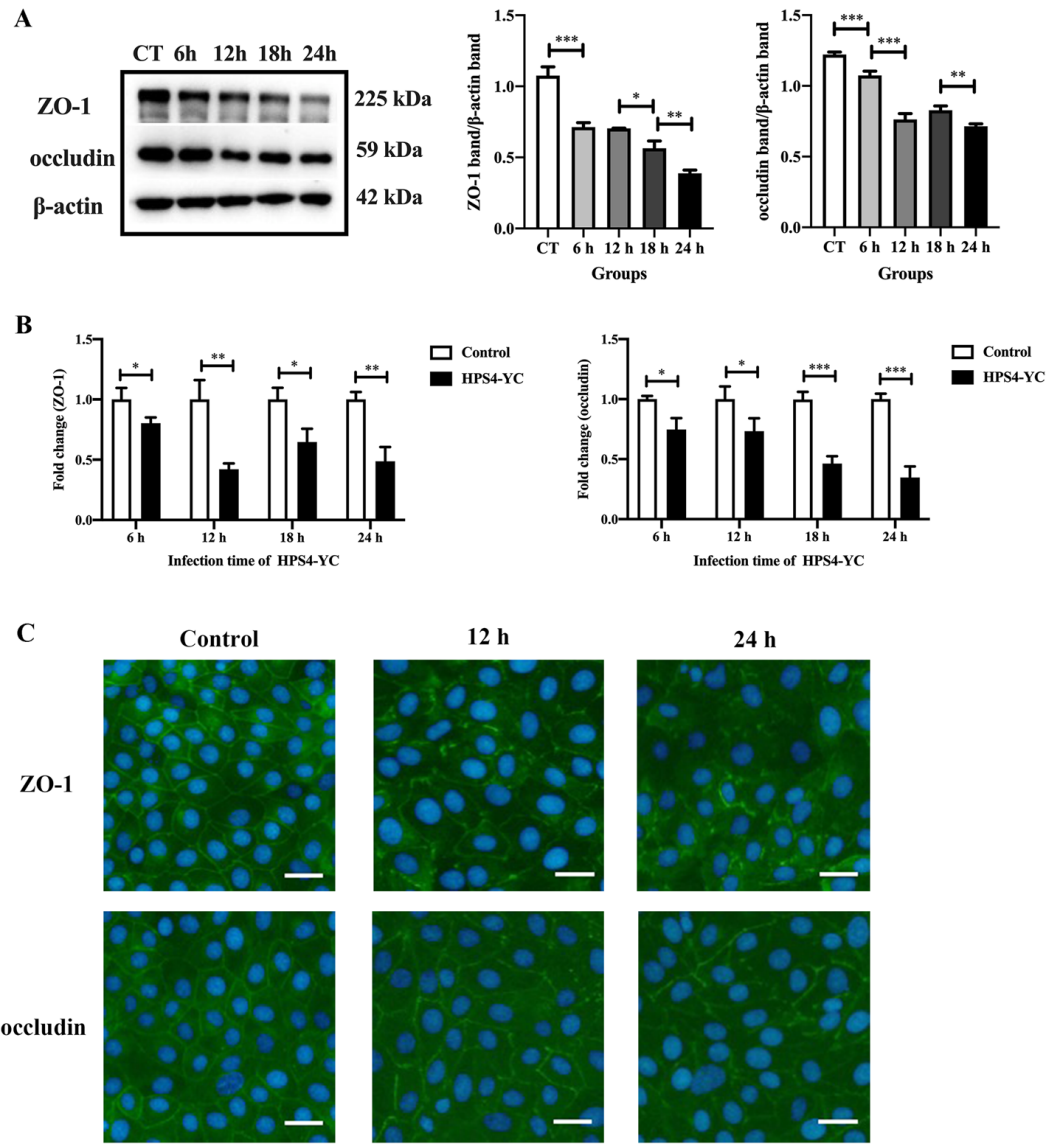
**HPS4-YC infection induced disruption of TJ in STEC.** STEC were infected with HPS4-YC for 6, 12, 18, and 24 h. **A** Protein levels of ZO-1 and occludin were analyzed by Western blot. The protein band intensities were quantified by ImageJ software. HPS4-YC caused a time-dependent decrease in tight junction protein levels in STEC. CT, the control group. **B** mRNA levels of ZO-1 and occludin were analyzed with qRT-PCR. The results are shown as the mean ± SD of triplicate independent experiments. Significant differences were analyzed using the Student *t* test. *, *P* < 0.05; **, *P* < 0.01; ***P < 0.001. **C** Immunofluorescence staining of ZO-1 and occludin in STEC. The infection times of HPS4-YC were 12 h and 24 h, then cells were fixed and the nucleus and TJ were stained (blue and green, respectively). HPS4-YC destroyed the integrity of TJ. Scale bar, 100 μm.

To explore the effect of HPS4-YC infection on the expression of TJ, STEC were infected with HPS4-YC for 6, 12, 18 and 24 h. Cellular protein was collected, and total RNA were extracted, followed by assessment by Western blotting and qRT-PCR. Western blot analysis shows that the expression of the TJ ZO-1 and occludin decreased during HPS4-YC infection (Figure 3A). Similarly, qRT-PCR results show that compared to the control group, HPS4-YC infection caused a significant downregulation in the mRNA levels of ZO-1 and occludin (Figure 3B). These results indicate that continuous HPS4-YC infection in STEC led to a reduction in ZO-1 and occludin.

The effect of HPS4-YC on TJ was further investigated by immunofluorescence staining of ZO-1 and occludin. As shown in Figure 3C, HPS4-YC infection had a destructive effect on epithelial cell tight junctions at 12 h and 24 h. Compared to the control STEC monolayers, the continuous tight junction structures of ZO-1 and occludin in HPS4-YC-infected cells were destroyed and could hardly constitute cell–cell junctions, which indicate that the integrity of the TJ ZO-1 and occludin were disrupted after HPS4-YC infection.

### HPS4-YC induced upregulation of proinflammatory cytokines through activation of MAPK and NF-κB signaling pathways

**Figure 4:**
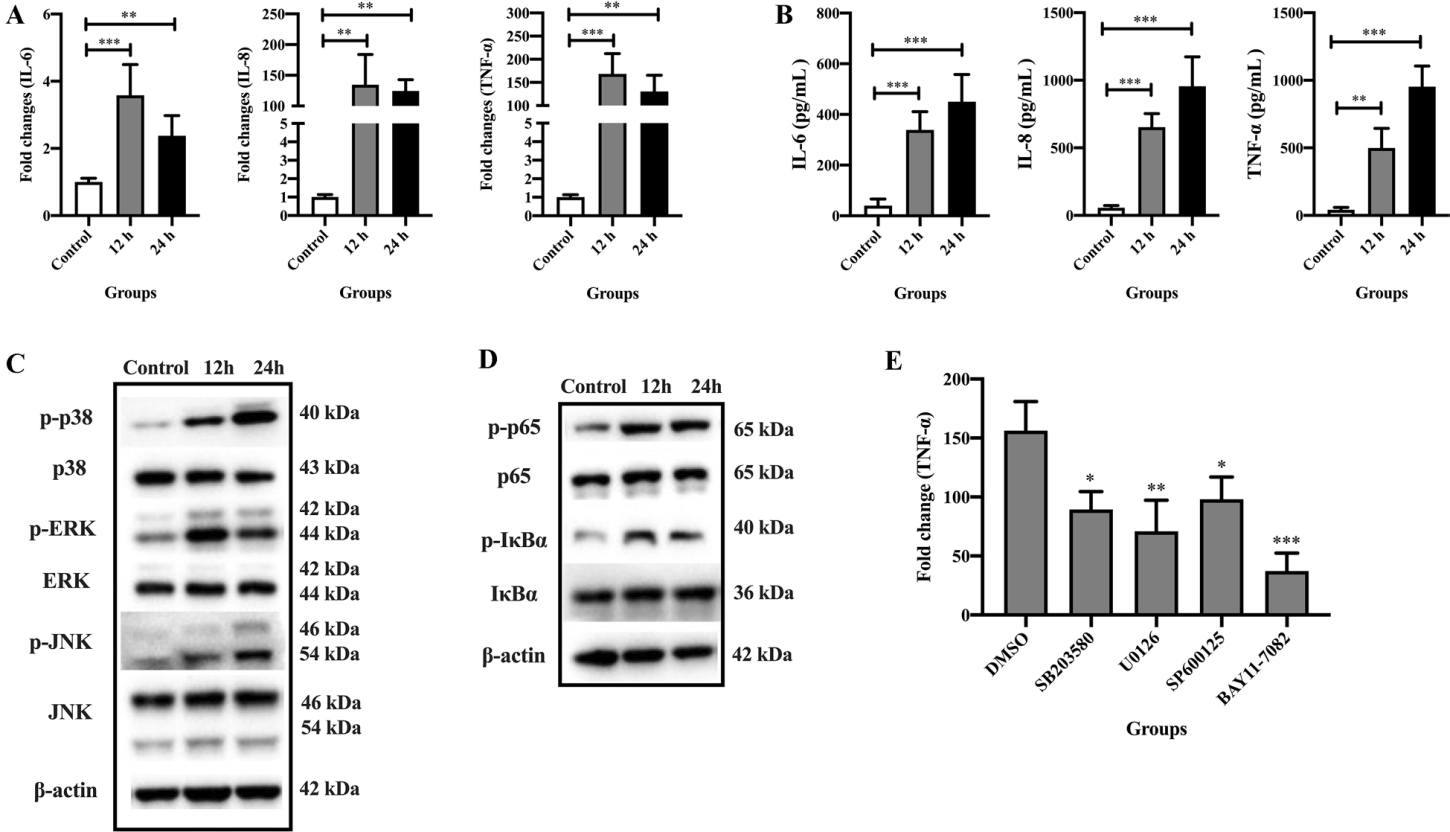
**Proinflammatory cytokines were upregulated through activation of MAPK and NF-κB signaling pathways in HPS4-YC-infected STEC.** **A** mRNA levels of cytokines IL-6, IL-8 and TNF-α were assessed through qRT-PCR assays. **B** Levels of the cytokines IL-6, IL-8 and TNF-α in culture supernatant of STEC were assessed by ELISA. **C**, **D** Activation of MAPK and NF-κB signaling pathways in STEC at 12 h and 24 h after HPS4-YC infection. The protein levels were determined by Western blot. **E** Expression of TNF-α was regulated by both MAPK and NF-κB signaling pathways. STEC were pretreated with DMSO or inhibitors for 1 h before HPS4-YC infection. The infection time of HPS4-YC was 12 h. SB203580, U0126, SP600125 and BAY11-7082 are inhibitors of p38, ERK, JNK and NF-κB, respectively. Data are shown as the mean ± SD of three independent experiments. Significant differences were determined using one-way ANOVA. *, *P* < 0.05; **, *P* < 0.01; ***P < 0.001.

The production of inflammatory cytokines is one of the important factors that affects TJ expression [23]. Total RNA from uninfected and HPS4-YC-infected STEC was extracted to assess the expression of the cytokines IL-6, IL-8 and TNF-α by qRT-PCR. The expression levels of the cytokines IL-6, IL-8 and TNF-α in the infected group were significantly higher than those in the control group at 12 h and 24 h post-infection (Figure 4A). Notably, the expression of IL-8 and TNF-α was upregulated hundreds of times at both 12 h and 24 h post-infection of HPS4-YC (Figure 4A). In addition, levels of cytokines IL-6, IL-8 and TNF-α in culture supernatant of STEC were evaluated. The ELISA results revealed that cytokines in the infected group were significantly higher than those in the control group at 12 h and 24 h, which was consistent with the result of qRT-PCR (Figure 4B).

MAPK and NF-κB are well-known signaling pathways regulating the expression of proinflammatory cytokines [24]. To determine whether HPS4-YC infection could activate MAPK and NF-κB signaling pathways, phosphorylation of p38, ERK, and JNK in the MAPK pathway and phosphorylation of p65 and IκBα in the NF-κB pathway were assessed by Western blotting. At 12 h and 24 h post-infection with HPS4-YC, the expression levels of p-p38, p-ERK, p-JNK, p-p65 and p-IκBα were all significantly upregulated (Figures 4C, D). These results indicate that HPS4-YC infection activated both MAPK and NF-κB signaling pathways.

In addition, the mRNA levels of TNF-α were selected to identify the pathways signaling cytokine production. When MAPK or NF-κB signaling pathways were inhibited in HPS4-YC-infected STEC, TNF-α expression was significantly decreased (Figure 4E), indicating that cytokine production in HPS4-YC-infected STEC was regulated by both MAPK and NF-κB signaling pathways.

### HPS4-YC-induced barrier damage was involved in the secretion of proinflammatory cytokines

**Figure 5:**
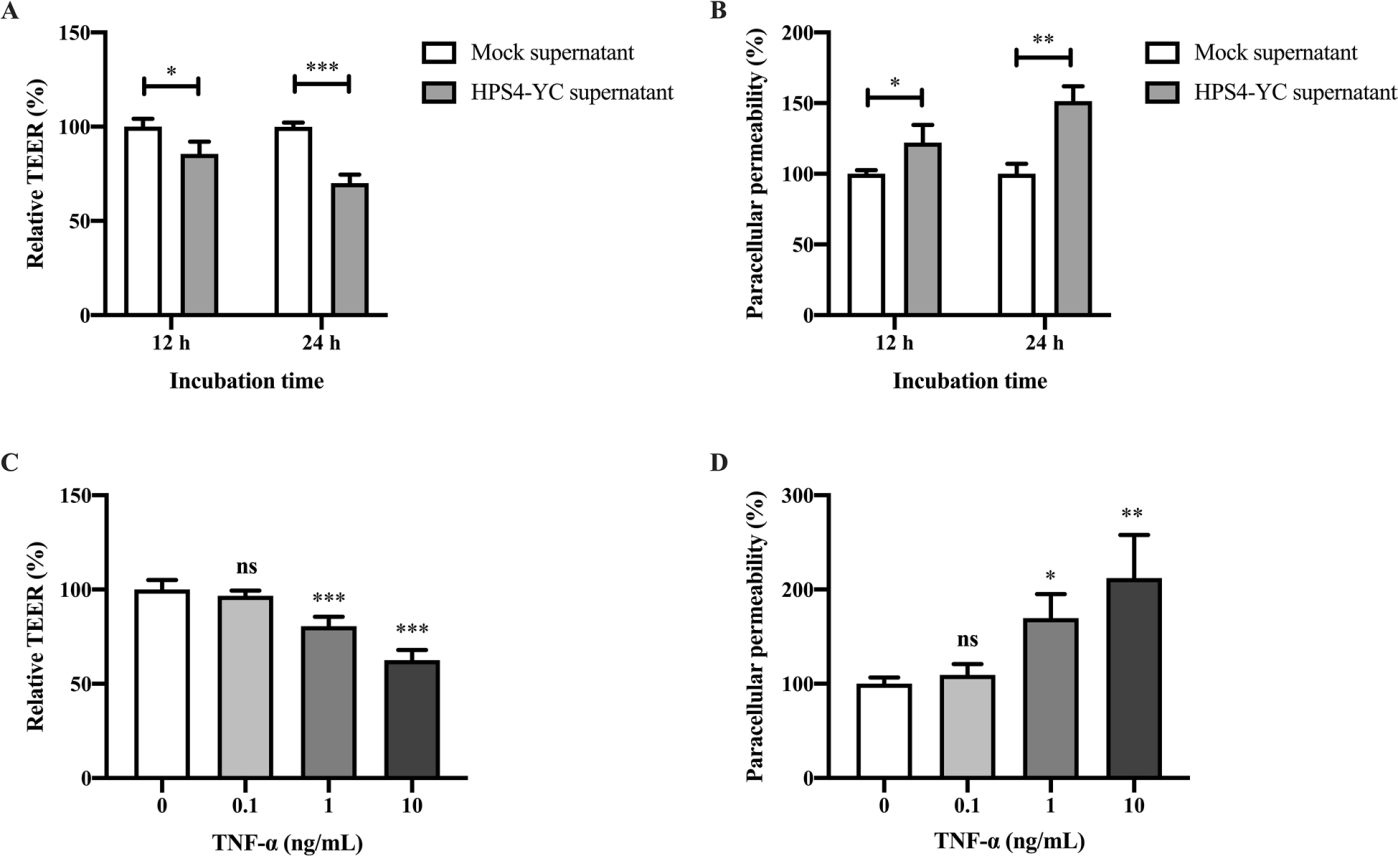
**Effect of inflammatory cytokines on swine tracheal epithelial barrier damage.** **A**, **B** Cytokines present in the supernatant decreased TEER and increased paracellular permeability respectively, promoting barrier damage. The supernatant of HPS4-YC-infected and mock-infected STEC was added to the upper chambers of transwells, and TEER and FITC-conjugated dextran crossing the transwell chamber were measured at 12 h and 24 h. **C**, **D** TNF-α damaged the epithelial barrier integrity. Different concentrations of TNF-α were added to the medium in the apical compartment for 24 h, and TEER and paracellular permeability were measured to determine their effect on epithelial barrier integrity. Data are shown as the mean ± SD of triplicate assays. Significant differences in A and B were determined using Student *t* test. Significant differences in C and D were determined using one-way ANOVA. ns: not significant; *, *P* < 0.05; **, *P* < 0.01; ***P < 0.001.

To investigate whether the damage to the tracheal epithelial barrier was a result of cytokines produced by epithelial cell infection with HPS4-YC, the supernatant of HPS4-YC-infected and mock-infected STEC was collected at 24 h post-infection. The supernatant was filtered to remove bacteria and then transferred to swine tracheal epithelial barrier model. Treatment with HPS4-YC supernatant resulted in a significant decline in TEER and increased the permeability of FITC-dextran (Figures 5A, B). Furthermore, recombinant human TNF-α was added to the apical chamber to determine its effect on the epithelial barrier. TNF-α treatment significantly decreased the TEER and increased the permeability of the epithelial barrier model (Figures 5C, D). As determined by CCK-8, TNF-α had no significant cytotoxicity in STEC during the experiment (data not shown). These results suggest that cytokines present in the cell supernatant after HPS4-YC infection contributed to epithelial barrier damage.

### HPS4-YC downregulated TJ in the lungs of piglets and caused a severe inflammatory response

**Figure 6:**
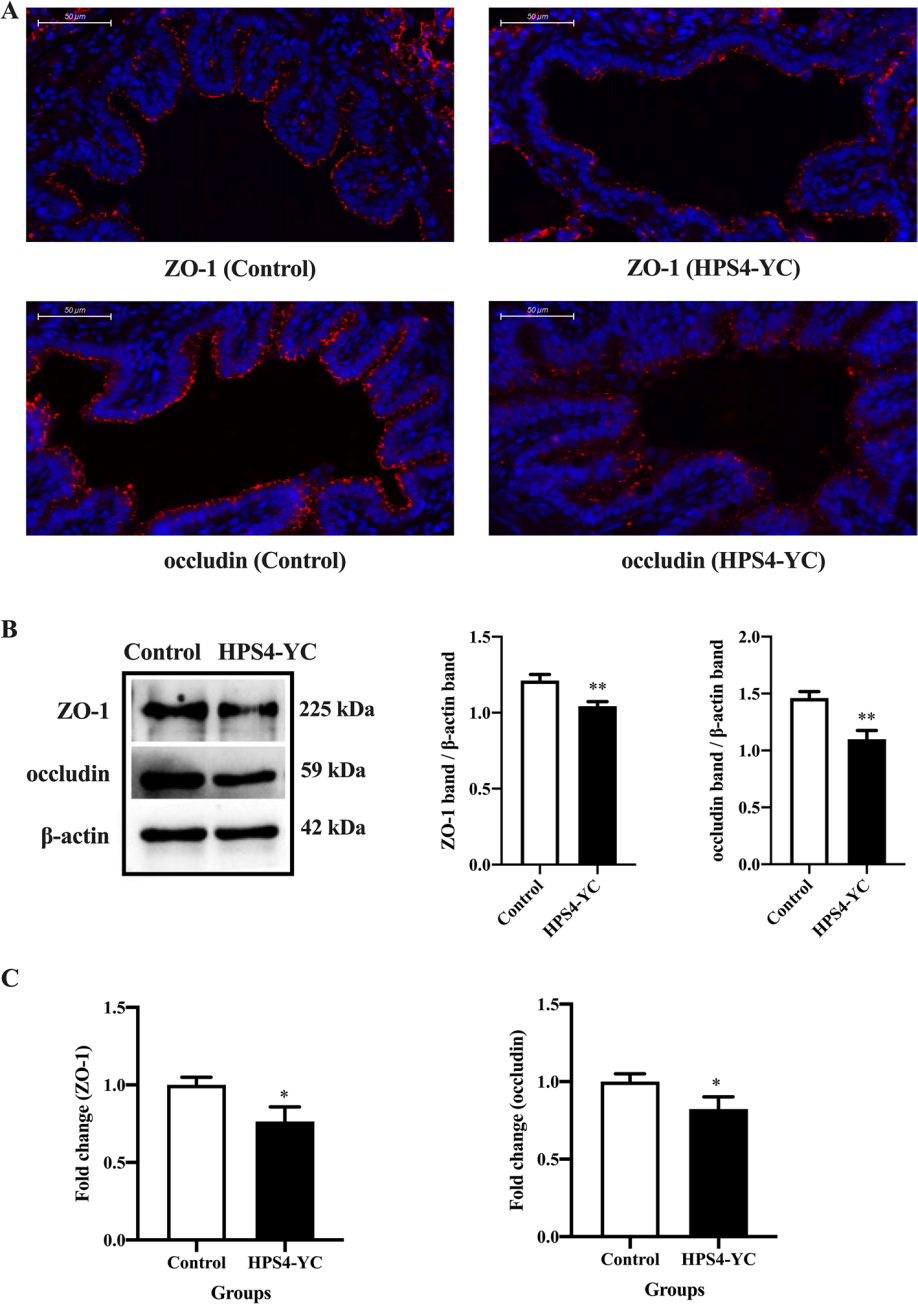
**HPS4-YC downregulated tight junction protein levels in the lungs of piglets.** **A** Immunofluorescence staining of ZO-1 and occludin in the lungs. Blue, the nucleus. Red, ZO-1 or occludin. Scale bar, 50 μm. **B** Protein levels of ZO-1 and occludin in the lungs were analyzed by Western blot. HPS4-YC decreased the expression of ZO-1 and occludin. **C** mRNA levels of ZO-1 and occludin in the lungs were analyzed with qRT-PCR. The results were in accordance with cell experiments. Data are shown as the mean ± SD of three independent experiments. Significant differences were determined using the Student *t* test. *, *P* < 0.05; **, *P* < 0.01.

Animal challenge experiments were performed to verify the disruption of TJ and the severe inflammatory response caused by HPS4-YC infection. The integrity of TJ in the lungs was observed through immunofluorescence assay; Western blotting and qRT-PCR were used to determine the expression of TJ. As shown in Figure 6A, ZO-1 and occludin in HPS4-YC-infected group were destroyed and discontinuous. In addition, the seronegative piglets may still carry pathogens, such as *G. parasuis*, but there is no TJ disruption in the control group, indicating that the disruption of TJ was caused by HPS4-YC infection. The protein levels were reduced compared with those in the control group, and the mRNA levels of ZO-1 and occludin in the lungs from the infected group were also lower than those from the control group (Figures 6B, C). These results suggest that HPS4-YC infection down-regulated the expression of TJ in the lungs, which was consistent with the results of our previous study.

**Figure 7:**
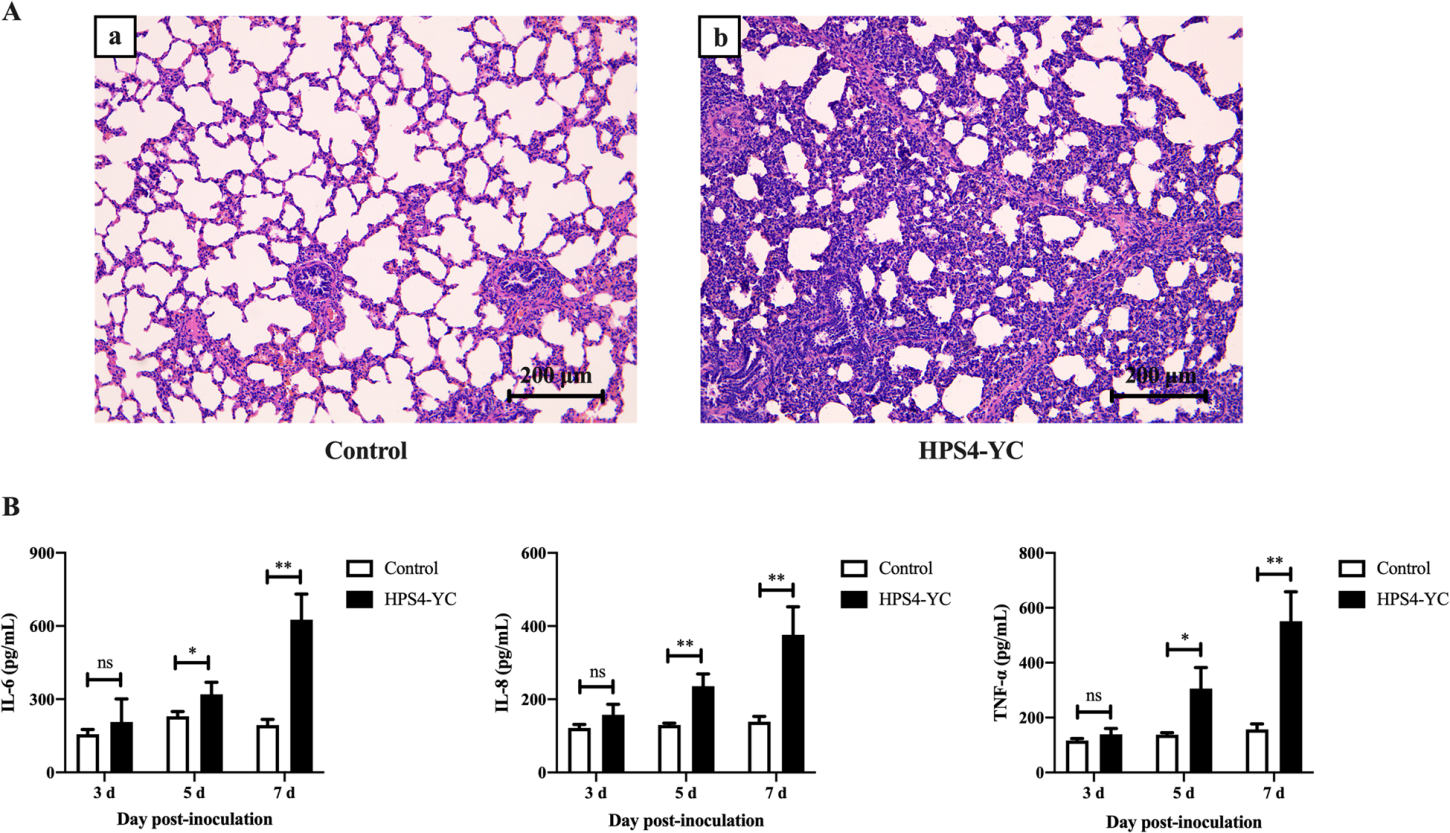
**Histopathological examination of the lungs and detection of proinflammatory cytokines in serum.** **A** Representative histopathological images of piglets mock-infected or infected with HPS4-YC. Scale bar, 200 μm. **B** Levels of the proinflammatory cytokines IL-6, IL-8 and TNF-α in serum samples were assessed by ELISA. Data are presented as mean ± SD of three independent experiments. Significant differences were determined using the Student *t* test. *ns* not significant; *, *P* < 0.05; **, *P* < 0.01.

In addition, H&E staining shows that the lungs exhibited alveolar wall thickening and inflammatory cell infiltration in the HPS4-YC-infected group (Figure 7A). Levels of cytokines IL-6, IL-8 and TNF-α in serum samples were measured at 3, 5 and 7 dpi. The ELISA results revealed that cytokines in the infected group were significantly higher than those in the control group at 5 dpi and 7 dpi (Figure 7B). HPS4-YC infection caused a severe inflammatory response in piglets.

## Discussion

*G. parasuis* is a commensal bacterium in the upper respiratory tract of pigs and can invade and cause disease [4]. However, how *G. parasuis* penetrates the respiratory barrier and causes infection is not yet fully understood. Epithelial cells are the first line of defense against pathogenic microorganisms, either respiratory or intestinal pathogens. Pathogenic microorganisms have developed different strategies to impair the epithelial barrier, resulting in invasive infection and systemic disease [25, 26]. TJ are located at the border of epithelial cells and are critical for maintaining epithelial barrier function [27]. Influenza A virus downregulates the expression of intercellular junction proteins E-cadherin, occludin and ZO-1, resulting in alveolar epithelial barrier damage [28]. The virulence factor pneumolysin of *Streptococcus pneumoniae* reduces the expression of ZO-1 in lung epithelial cells and causes a breakdown of the epithelial barrier [29]. Here, we demonstrate that HPS4-YC downregulated the expression of TJ ZO-1 and occludin, breaking the integrity of the swine tracheal epithelial barrier and enabling rapid bacterial translocation.

The assembly, disassembly and maintenance of TJ are influenced by various physiological and pathological stimuli. Microbial agents can modulate signaling pathways that participate in the organization of TJ, such as the MLCK and MAPK pathways, leading to changes in gene expression encoding TJ or cellular redistribution of TJ [26, 30]. In addition, altered epithelial TJ have been reported to result from proinflammatory cytokine production, which can also subsequently activate regulatory pathways linked to TJ [26]. Here, we show that HPS4-YC induced significant upregulation of IL-6, IL-8 and TNF-α. Inflammation is a double-edged sword; although it is necessary for relieving of infection, an excessive inflammatory response can cause tissue damage and promote infection [31]. *Staphylococcus aureus* triggers the secretion of IL-6 and IL-8, leading to human nasal epithelial barrier dysfunction [7]. *Klebsiella pneumoniae* induces IFN-γ, causing a decrease in airway epithelial barrier integrity [32]. Consistent with these observations, TNF-α production in HPS4-YC-infected STEC increased paracellular permeability and contributed to epithelial barrier disruption. However, in contrast to the above studies, Sajjan et al. reported that rhinovirus-induced disruption of airway epithelial barrier function is independent of proinflammatory cytokines [33]; a strong proinflammatory response induced by influenza virus is not responsible for alveolar epithelial barrier damage [10]. The destructive effect of inflammatory cytokines on the epithelial barrier is one of the ways that facilitates microbial pathogen infection, and this effect might be related to the type of epithelial cells, the levels of cytokines and the presence of anti-inflammatory cytokines such as IL-10 [15].

Additionally, the signaling pathways that regulate cytokine production in STEC were investigated. Our data show that HPS4-YC induced the production of TNF-α through activation of both the MAPK and NF-κB signaling pathways, which was consistent with previous studies about *G. parasuis* [34, 35]. Previous studies have reported that TNF-α induce increased intestinal epithelial or endothelial barrier permeability through NF-κB activation [36, 37]. Accumulating evidence suggests that the MAPK pathway can regulate the expression of TJ in different types of epithelial cells and the permeability of the epithelial barrier [38]. For example, activation of ERK/MAPK leads to decreased ZO-1 and occludin expression in H1N1 influenza virus-infected A549 cells [28]. Clarke et al. reported that *Streptococcus pneumoniae* and *Haemophilus influenzae* downregulate the expression of TJ in respiratory epithelial cells and destroy the integrity of the epithelial barrier through activation of the p38/MAPK pathway [11]. These observations suggest that in addition to the production of cytokines, the MAPK pathway may directly regulate TJ expression during HPS4-YC infection. There are several unsolved issues in our study, such as the interactions between *G. parasuis* effectors and surface receptors that lead to activation of MAPK and NF-κB signaling pathways, which have not been investigated. Whether and how MAPK and NF-κB activation in HPS4-YC-infected STEC results in a reduction of TJ require further research.

In summary, we have shown that *G. parasuis* infection downregulated TJ and disrupted epithelial barrier integrity, favoring bacterial translocation. The disruption of TJ and epithelial damage were related to the release of proinflammatory cytokines, and the expression of cytokines depended on the activation of MAPK and NF-κB. These findings will help clarify the pathogenic mechanism of *G. parasuis*, and contribute to better control strategies of swine Glässer disease.

## Abbreviations

*G. parasuis*: *Glaesserella parasuis*
GPS4: *Glaesserella parasuis* Serotype 4
MOI: multiplicity of infection
TJ: tight junction proteins
STEC: swine tracheal epithelial cells
TEER: transepithelial electrical resistance
MAPK: mitogen-activated protein kinases
NF-κB: nuclear factor-kappa B
dpi: day post-inoculation
SDS-PAGE: sodium dodecyl sulfate-polyacrylamide gel electrophoresis
PVDF: polyvinylidene fluoride
qRT-PCR: quantitative reverse transcription PCR
IL: interferon
TNF: tumor necrosis factor
PCV2: porcine circovirus type 2
PRRSV: porcine reproductive and respiratory syndrome virus
